# Personalizing symptom monitoring and contact tracing efforts through a COVID-19 web-app

**DOI:** 10.1186/s40249-020-00711-5

**Published:** 2020-07-13

**Authors:** Kevin Yi-Lwern Yap, Qihuang Xie

**Affiliations:** 1 Department of Public Health, School of Psychology and Public Health, La Trobe University, Melbourne (Bundoora), Victoria 3086 Australia; 2 Department of Pharmacy, Faculty of Science, National University of Singapore, Block S4A, Level 2, 18 Science Drive 4, Singapore, 117543 Singapore

**Keywords:** COVID-19, Novel coronavirus, Symptom monitoring, Contact tracking, Web-app

## Abstract

**Background:**

The Coronavirus Disease 2019 (COVID-19) pandemic has sparked attention in many countries, especially those that have experienced a steep spike in the number of identified cases. The continued spread of the coronavirus suggests that this situation may be here to stay for a while. Contact tracing is a time-consuming and resource-intensive process, which taps on the already lean healthcare resource in certain countries. Furthermore, the massive infodemic on COVID-19 on the Internet has also resulted in widespread circulation of misinformation online. This outbreak has evoked irrational fear and anxiety from the public, which has resulted in destabilizing of societal norms, such as panic buying and hoarding of daily necessities, and can potentially pose serious health risks to the public. The aim of this paper is to present a *COVID-19 Symptom Monitoring and Contact Tracking Record* (CoV-SCR) web-app as a bottom-up, proactive approach to supplement the current management strategies for COVID-19.

**Main text:**

The CoV-SCR web-app (http://bit.ly/covscrapp) enables individuals to keep a personal record of their close contacts and monitor their symptoms on a daily basis, so that they can provide relevant and accurate details when they see the doctor and during the contact tracing process. Individuals can record their temperature and rate their symptoms on a 5-point severity scale, as well as record details of their travel and contact history for the last 14 days. The recorded information will be sent to their e-mail address for potential symptom monitoring and contact tracing purposes. In addition, this web-app consolidates evidence-based information on the coronavirus from credible sources, such as the World Health Organization, countries’ health authorities, and PubMed literature.

**Conclusions:**

A COVID-19 Symptom Monitoring and Contact Tracking web-app has been developed to facilitate contact tracing efforts through public engagement. This app serves an additional purpose of providing information about COVID-19 from reliable resources.

## Background

Coronavirus Disease 2019 (COVID-19) has spread with unprecedented speed and scale and has since affected over 188 countries. As of 5th June 2020, the total number of confirmed cases worldwide has exceeded six million and over 390 000 people have died from the disease [1]. Although it has been reported that the number of newly confirmed cases and deaths has slowed in China, the sudden spike in the number of cases in other parts of the world is a cause for concern. Outside of Asia, the virus has made its way to Europe, North and South America, Middle East, and Australia — causing emergency lockdowns of overseas travel. The sudden surge in the number of confirmed cases worldwide suggests that all countries should stay vigilant, step up disease control measures and deploy a more concerted multisectoral approach to contain the spread of the virus. The aim of this paper is to present a *COVID-19 Symptom Monitoring and Contact Tracking Record* (CoV-SCR) web-app to supplement the current management strategies for COVID-19.

## Main text

Collecting accurate epidemiological data through contact tracing can be an effective strategy to promote situational awareness and devise effective interventions to prevent and control the outbreak within a country. However, contact tracing is a time-consuming and resource-intensive process. Furthermore, contact tracing through interviews are prone to recall bias as individuals may not necessarily recall events that have occurred 14 days ago accurately. In order to reduce the resources needed for contact tracing, China deployed several methods, such as facial recognition and collecting data from railway and airline companies, to track the movement of its citizens [2]. However, this model cannot be replicated in other countries due to differences in social environments and privacy concerns.

**Fig. 1:**
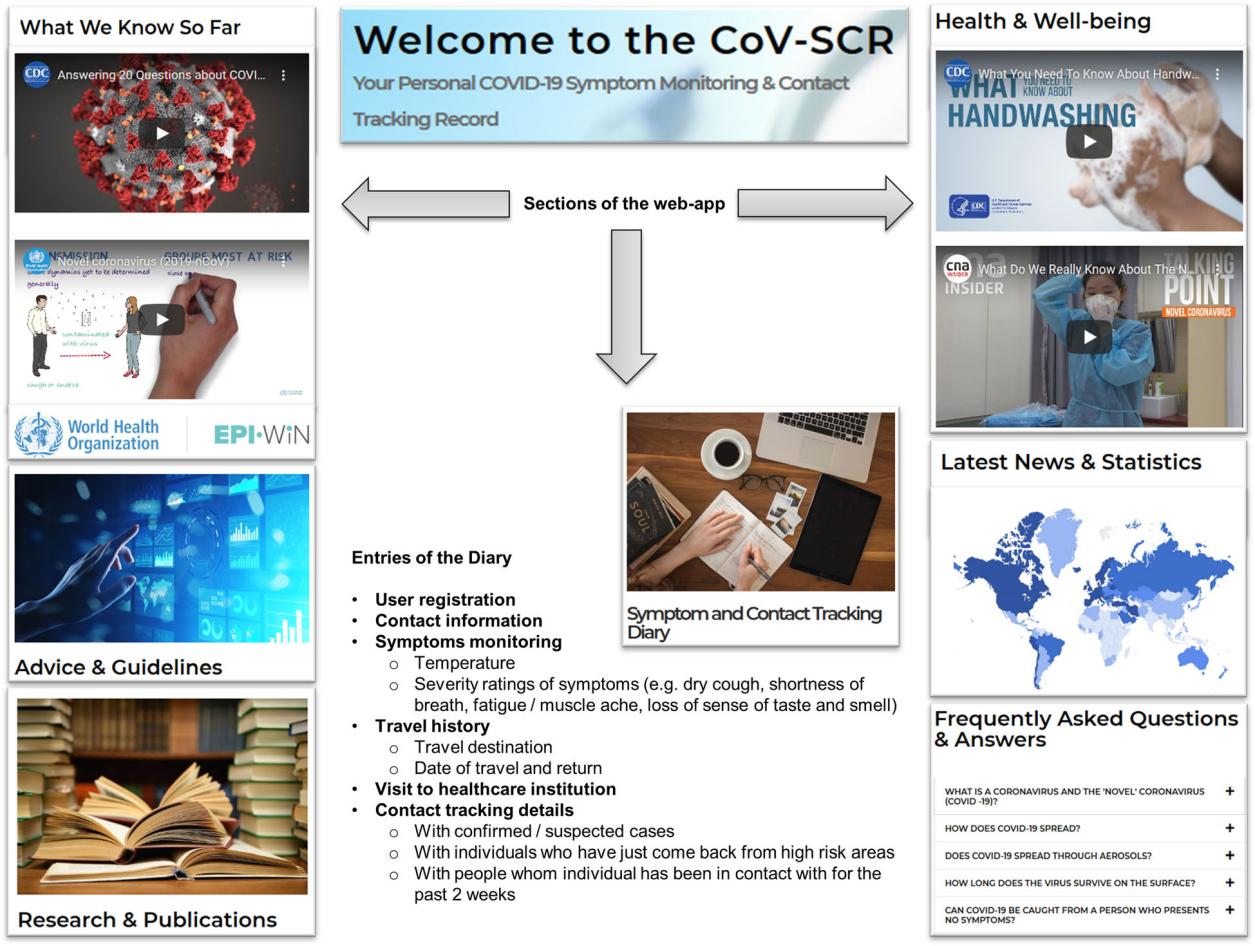
Screenshots showing the sections of the COVID-19 Symptom Monitoring and Contact Tracking Record (CoV-SCR) web-app. COVID-19: Coronavirus Disease 2019

In addition to the current top-down, reactive approach of contact tracing initiated by health authorities, the CoV-SCR web-app (http://bit.ly/covscrapp) (Fig. 1) acts as a bottom-up, proactive approach that enables individuals to keep a personal record of their close contacts and symptoms on a daily basis, so that they can provide relevant and accurate details when they see the doctor and during the contact tracing process. People who feel the need to monitor themselves and their families/close contacts during this period can record their temperature and rate their symptoms on a 5-point severity scale. In addition, users can record details of their travel and contact history for the last 14 days. The recorded information will be sent to their email address, which can be used for potential symptom monitoring and contact tracing purposes. The act of recording these details manually enable the user to be more cognizant of their own risk of exposure to COVID-19. Should the user find out that they have come into close contact with a suspected or confirmed case, they can alert third-party contacts that they have recorded (e.g. friends and colleagues whom they have been in contact with) to seek medical attention and take precautionary measures such as voluntary self-quarantine/self-isolation or taking social distancing measures and wearing face masks to minimize contact and transmission. Additionally, third-party contacts can notify other close contacts whom they have recorded as well, thus resulting in a positive feedback loop through a ripple effect, which will help in controlling the spread of COVID-19. This process will greatly benefit individuals, especially those who have susceptible populations at homes, such as children and the elderly, as it gives them a systematic way to monitor themselves and their families, analyze their daily activities and seek immediate medical attention, if necessary. Furthermore, this method of recording not only reduces the amount of healthcare resources needed, but also increases the reliability of contact tracing and can be used as a form of early identification of suspected cases, which then allows the individual or health authorities to carry out interventions early (e.g. need for isolation and/or medical treatment). Healthcare professionals/health authorities, with the consent from the user, will be able to access their symptoms and close contacts through the emails sent by the web-app, rather than relying on the user’s memory. In order to address privacy concerns, the privacy policy of the web-app clearly states the information is collected from users and how this information is used and stored. Furthermore, disclosure of personal information through the web-app records is purely at the discretion by the user. Users can easily access their recorded entries through their registered email inbox. The data collected at the backend database will only be used for aggregate data analysis and educational purposes.

Over the past few months, countries have started to employ digital contact tracing apps in the fight against COVID-19. However, even though these contact tracing apps may be efficient and convenient, they have their limitations. For digital contact tracing apps that use Bluetooth technology, they can detect signals up to 10 m [3]. This means people who are far apart from one another (more than 1 m) may be identified as being in close contact by such apps. Furthermore, Bluetooth signals can penetrate walls [4], hence individuals who are blocked in between by a wall (e.g. in two adjacent rooms) can also be identified as being in close contact. On the other hand, apps that use Global Positioning Systems (GPS) for location-tracking may be worse than Bluetooth at measuring proximity indoors, as GPS technologies do not work well indoors and cannot identify which floor a person is at within a building [5]. The limitations of Bluetooth and GPS technologies can lead to false positive and false negative results, which undermine their effectiveness for contact tracing. Thus, the CoV-SCR web-app is useful as an additional public utility tool to supplement the current contact tracing apps to enhance the tracking of community transmission of COVID-19 by allowing users to manually record their close contact information in a more personalized way.

Besides the social impact caused by COVID-19, the public is also struggling to fight a “massive infodemic” of an overabundance of information about this pandemic, which includes inaccurate news, making it difficult for people to differentiate which pieces of information are factual and evidence-based. As such, various governments and organizations have invested in substantial resources to deal with this rapid spread of information online. Although wearing face masks have shown to be effective in controlling the spread of COVID-19 [6], and this practice has been encouraged by various governmental organizations and the World Health Organization (WHO), the irrational fear and anxiety fuelled by the infodemic at the start of the COVID-19 pandemic have led to people stockpiling face masks and other personal protective equipment [7]. This resulted in a shortage of supplies for healthcare professionals. Furthermore, behaviors such as panic buying and hoarding of food and daily necessities have been prevalent [8]. The proliferation of fake news online, for example, preventing COVID-19 infection through garlic consumption, eradicating the coronavirus by spraying alcohol and chlorine over the body, and reusing face masks after washing or cleaning with sanitizers [9], may potentially pose serious health risks if left unchecked. As such, the WHO is working to clamp down on disinformation, as well as provide the public with access to timely and accurate information on COVID-19 [10]. In addition to the symptom monitoring and contact tracking features, the CoV-SCR web-app also aims to combat the infodemic by consolidating information on the coronavirus pandemic from credible sources, such as the WHO, countries’ health authorities, and PubMed literature, which will be regularly updated. This information is meant for the public and is organized into general information and frequently asked questions; latest news and statistics; ways to maintain health and well-being; advice and guidelines; and relevant research publications.

## Conclusions

With the massive infodemic surrounding COVID-19 and the need for worldwide symptom monitoring and contact tracing, the CoV-SCR web-app can help provide evidence-based information and advice through credible sources, as well as alleviate the resources needed for public health efforts to contain the disease, by enabling the public to contribute proactively through regular personalized symptom monitoring and tracking of close contacts.

## Data Availability

Not applicable.

## Abbreviations

COVID-19: Coronavirus Disease 2019
CoV-SCR: COVID-19 Symptom Monitoring and Contact Tracking Record
